# The depletion of ubiquilin in *Drosophila melanogaster* disturbs neurochemical regulation to drive activity and behavioral deficits

**DOI:** 10.1038/s41598-020-62520-y

**Published:** 2020-03-30

**Authors:** Salinee Jantrapirom, Yosuke Enomoto, Jirarat Karinchai, Mizuki Yamaguchi, Hideki Yoshida, Eiichiro Fukusaki, Shuichi Shimma, Masamitsu Yamaguchi

**Affiliations:** 1 Department of Pharmacology, Faculty of Medicine, Chiang Mai University, Chiang Mai, 50200 Thailand; 2 Department of Applied Biology, Kyoto Institute of Technology, Matsugasaki, Sakyo, Kyoto 606-8585 Japan; 3 Department of Biotechnology, Graduate School of Engineering, Osaka University, 2-1 Yamadaoka, Suita, Osaka 565-0871 Japan; 4 Department of Biochemistry, Faculty of Medicine, Chiang Mai University, Chiang Mai, 50200 Thailand

**Keywords:** Neurodegeneration, Amyotrophic lateral sclerosis

## Abstract

*Drosophila melanogaster* is a useful and highly tractable model organism for understanding the molecular mechanisms of human diseases. We previously characterized a new *dUbqn* knockdown model that induces learning-memory and locomotive deficits mediated by impaired proteostasis. Although proteinopathies are the main causes of neurodegenerative diseases, limited information is currently available on the relationship between proteostasis and neurodegenerative-related behavioral perturbations, such as locomotion, wakefulness, and sexual activities. Thus, the present study aimed to elucidate the mechanisms by which *dUbqn* depletion which is known to cause proteinopathies, affects neurodegenerative-related behavioral perturbations. Pan-neuronal *dUbqn*-depleted flies showed significantly reduced evening activity along with altered pre- and postsynaptic structural NMJ’s proteins by attenuating signals of Bruchpilot puncta and GluRIIA clustering. In addition, the neurochemical profiles of GABA, glutamate, dopamine, and serotonin were disturbed and these changes also affected courtship behaviors in *dUbqn*-depleted flies. Collectively, these results extend our understanding on how *dUbqn* depletion affects neurochemical regulation to drive behavioral disturbances that are generally found in the early stage of neurodegenerative diseases. Moreover, the present study may contribute a novel finding to the design of new agents that prevent disease progression or even treat diseases related to neurodegeneration.

## Introduction

An effective regulation of proteostasis is a fundamental requirement for cells, particularly neurons, which are highly susceptible to proteotoxicity. Disruptions in protein turnover by stressors, gene mutations, or even a normal aging process have been shown to accelerate the development of proteinopathy-related disorders, such as Alzheimer’s disease (AD), Parkinson’s disease (PD), Amyotrophic Lateral Sclerosis (ALS), and Frontotemporal Dementia (FTD)^1–3^. Neuronal-related symptoms, including motor, cognitive, and behavioral alterations, manifest in the early stages of neurodegenerative progression^4,5^. However, the mechanisms underlying these early symptoms have not yet been elucidated in detail.

Ubiquilins (UBQLNs), an important group of the proteins involved in proteostasis, has been extensively studied in a wide spectrum of neurodegenerative disorders. They are characterized as Ubiquitin-like (UbL)-Ubiquitin-associated (UbA) proteins that recognize and bind to ubiquitylated substrates in order to target them for degradation via proteasomes, endoplasmic reticulum-associated protein degradation (ERAD), and macroautophagy^6–9^. The five human *UBQLNs* homologues such as *UBQLN1, UBQLN2, UBQLN3, UBQLN*4 and *UBQLNL* have been established so far. However, only mutations in *UBQLN2* and *UBQLN4* strongly associate to ALS or ALS/FTD. Besides, UBQLNs also exhibit several distinctive activities, such as an actin and intermediate filament association, cell survival regulation by coordinating with protein-disulfide isomerase (PDI), and their emerging role in driving gene transcription by dUbqn-dHP1c-WOC-ROW complexes^10–12^.

*Drosophila melanogaster* is a useful and highly tractable model organism for studying and understanding the molecular mechanisms of human diseases due to biologically and physiologically highly conserved pathways between *Drosophila* and humans. *Drosophila* has a single homologue of *UBQLN* in its genome called *dUbqn*, which shows high similarity to human *UBQLN1, 2*, and *4* in the major domains as well as functions^13^.

Bedford *et al*. discovered the regulatory function of UBQLNs in synapses to facilitate GABA-A receptor expression^14^. Moreover, the dysregulation of dUbqn also contributes to defects in postsynaptic growth and synaptic proteostasis^15^. Although previous studies reported a role for UBQLNs in receptor regulation at synapses, its involvement in the control of neurochemical expression remains unclear. Dysfunctional UBQLNs with or without mutations cause ubiquitylated protein accumulation in the early stages of neurodegenerative diseases^16^. At the same stage, declines in motor-cognitive functions and behavioral changes, such as the loss of sexual abilities and sleep-awakening disturbances, are generally observed before the loss of affected neurons^5,16–18^. Therefore, the mechanisms by which dysfunctional UBQLNs affect neurochemical changes to induce motor, cognitive, and behavioral deficits are interesting to investigate in more detail.

Recently, our group took advantage of the novel mass spectrometry imaging (MSI) method to visualize neurochemicals inside the body of *Drosophila*^19^. In addition, with a newly established *dUbqn* knockdown model we demonstrated that proteostasis impairments can drive learning-memory and locomotive deficits^20^. Thus, we intend to use the *dUbqn* knockdown model to gain insights into the changes in more complex behaviors including copulation during proteostasis impairments through investigations on neurochemical regulation.

## Materials and Methods

### *Drosophila* handling and stocks

All flies were cultured at 25 °C under humidity-controlled conditions. Standard *Drosophila* food contains 0.65% agar, 10% glucose, 4% dry yeast, 5% corn flour, and 3% rice powder^19,20^. Flies carrying *UAS-dUbqnIR*_107-494_ (106050) and *UAS-dUbqnIR*_471-824_ (47447) and *UAS-empty vector* (60100) from the Vienna *Drosophila* Resource Center (VDRC), *w*^1118^ (108479) from Kyoto stock center and those carrying *elav-GAL4* (41500) and *UAS-GFP-IR* (41550) from the Bloomington *Drosophila* Stock Center (BDSC) were used in the present study. To minimize the effects of the genetic background, flies were backcrossed 6 times with the *w* strain before experiments.

### *Drosophila* activity assay

Newly eclosed adult male flies carrying *UAS*-*dUbqnIR*_107-494_/+;*elav-GAL4/*+, +;*elav-GAL4/UAS-dUbqnIR*_471-824_ and *UAS*-*GFP-IR*/+;*elav-GAL4*/+ were selected under CO_2_ anesthesia. Three-day-old flies from each strain were individually placed in *Drosophila* activity monitoring tools (Trikinetics, Waltham, MA, USA) housed inside an incubator controlled at 25 °C. Fly activity was monitored for 7 days under a 12-h light-dark cycle^21^. The amount of activity by each fly was recorded every 30 min.

### *Drosophila* courtship assay

Pairs (virgin male and virgin female) of 3-day adult flies carrying *UAS*-*dUbqnIR*_107-494_/+;*elav-GAL4*/+, +;*elav-GAL4/UAS-dUbqnIR*_471-824_ and *UAS*-*GFP-IR*/+;*elav-GAL4*/+ were selected under CO_2_ anesthesia. Briefly, flies were allowed to recover from anesthesia for at least 1 h, and an aspirator was then used to gently transfer an individual male to the courtship chamber followed by a female^22^. Recordings of behavior were immediately started and conducted for at least 20 minutes.

### Neuromuscular junctions (NMJs) visualization

Third instar larvae carrying *w*^1118^*, UAS*-*dUbqnIR*_107-494_/+;*elav-GAL4*/+, +;*elav-GAL4/UAS-dUbqnIR*_471-824_*, Empty vector*/+;*elav-GAL4*/+ and *UAS*-*GFP-IR*/+;*elav-GAL4*/+ were dissected in HL3 saline and fixed in Bouin’s solution at 25 °C for 30 minutes. Larval NMJs were incubated in 2% bovine serum albumin and 0.1% Triton-X 100 in PBS at 25 °C for 30 minutes. Fluorescein isothiocyanate (FITC)-conjugated anti-horseradish peroxidase (HRP) IgG (1:1000) was used as the detection antibody^20^. Primary antibodies, such as mouse monoclonal anti-Disc large (Dlg) IgG (1:300), mouse monoclonal anti-Bruchpilot (Brp) IgG (1:50) and anti-GluRIIA IgG (1:80), were used to visualize active zones (AZ) under 4 °C in a 16-hour incubation. Samples were incubated with secondary antibodies labeled with Alexa 594 (1:400) before mounting with Vectashield (Vector Laboratories, Burlingame, CA, USA). Confocal microscopic images were taken with a confocal laser scanning microscope (Fluoview FV10i, Olympus, Tokyo, Japan) and processed with ImageJ software^21^.

### Larvae tissue preparation

For sample treatment, third instar larvae at similar developmental stages (within 6 hours difference) were picked and dipped in 70% ethanol before embedding. Then, they were embedded in 10% gelatin on base-mold (7 mm × 7 mm × 5 mm; FALMA, Tokyo, Japan)^23,24^. Gelatin powder (Sigma-Aldrich, St. Louis, MO, USA) was dissolved in ultrapure water (Genpure UV-TOC xCAD PLUS, Thermo Fisher Scientific, Waltham, MA, USA) and mixed for 10 min at 42 °C, using a thermomixer (Eppendorf, Hamburg, Germany) until a homogeneous solution was obtained^24^. After aligning the larvae per their body axes in the embedding material, the molds were frozen rapidly in liquid nitrogen. The frozen blocks of gelatin with flies were placed in cryomicrotome (CM1950, Leica, Wetzlar, Germany) at −20 °C for 1 hour^24^. These blocks were then placed on a tissue-holder, fixed with an optimum cutting temperature polymer (Leica, Wetzlar, Germany), and sliced at 30-μm thickness. The tissue sections were thaw-mounted onto Indium-tin-oxide (ITO)-coated glass slides (100 Ω/m^2^ without anti-peeling coating, Matsunami Glass, Osaka, Japan) by warming them up by placing a finger on the posterior side of the glass slides^24^. The glass slides were then dried at 40 °C for 5 min and placed in 50-mL conical tubes containing silica gel^24^.

### On tissue derivatization

2,4-diphenyl-pyranylium tetrafluoroborate (DPP-TFB) (Sigma-Aldrich, St. Louis, MO, USA) was dissolved in methanol to prepare 10 mg/mL stock solutions^23^. DPP-TFB solutions used for derivatization contained 6 µL of the stock solution, 69 µL of 60% methanol, and 1 µL of triethylamine. After sectioning, on-tissue derivatization was performed with 50 µL of DPP-TFB solution deposited onto each section manually using an airbrush (PS-270, GSI Creos, Tokyo, Japan)^23^. Then the tissue sections were incubated for 90 minutes at 25 °C to facilitate the derivatization reaction.

### Matrix application

2,5-dihydroxybenzoic acid (DHB) (Sigma-Aldrich, St. Louis, MO, USA) was applied to tissue sections via spray coating method^24^. DHB solution (50 mg/mL dissolved in 50% methanol) was applied to each section using an airbrush (PS-270, GSI Creos, Tokyo, Japan)^24^. The airbrush was maintained at 10 cm distal from glass slides and an approximately 50 µL DHB solution was sprayed to each section and then completely dried. The spray-dry process was repeated multiple times^24^.

### Mass spectrometry imaging (MSI) for neurochemicals

MSI was performed using an iMScope TRIO (Shimadzu, Kyoto, Japan). Both optical images and ion distribution were obtained within the same instrument under atmospheric pressure^24^. Nd:YAG laser (λ = 355 nm, 1 kHz) was used as the MALDI laser source, and laser irradiation was repeated 150 times on each data point with a laser power of 45.0 (arbitrary unit in iMScope TRIO)^24^. The voltages of the sample stage and detector were 3.50 kV and 2.10 kV, respectively^24^. A positive ion detection mode was used with an *m/z* range of 200–400 for detection of neurochemicals. Tandem mass spectrometry (MS/MS) analysis was carried out to visualize DPP-derivatized GABA, glutamate, dopamine, and serotonin with the selected precursor *m/z* 318, 362, 368, and 391. The ion peak in the product ion mass spectra provided the peak at *m/z* 232.11, therefore the peak intensity of *m/z* 232.11 was visualized for neurochemicals imaging. The IMAGEREVEAL (Shimadzu, Kyoto, Japan) was used for the data analyses.

### Sample preparation for the Liquid chromatography-tandem mass spectrometry (LC-MS/MS) analysis

Five third instar larvae at similar developmental stages in 1.5-mL Eppendorf tubes were freeze-dried for 12 h using a freeze dryer (VD-800F, Taitec, Tokyo, Japan). Freeze-dried samples were milled using a ball mill (Retsch, Haan, Germany) at 20 Hz for 1 min. Five hundred microliters of extraction solvent (methanol/water/chloroform= 5:2:2 v/v/v%) and 20 µL of 1 mM 3,4-dihydroxybenzylamine as an internal standard were added to each tube with the ball-milled sample. The sample was mixed using a vortex and sonicated for 1 minute. After sonication, the sample was centrifuged at 10000×*g* at 4 °C for 10 minutes. After centrifugation, 400 µL of the supernatant was transferred to a new tube containing 150 µL of water. Then the sample was mixed by vortexing and centrifuged at 10000×*g* at 4 °C for 10 minutes to separate the polar and non-polar phases. In the GABA, glutamate, and dopamine analysis, the upper polar phase was diluted by 10-fold using water with 0.1% formic acid and syringe filtration (0.2-µm PTFE hydrophilic membrane, Merck) was performed. The filtered solution was transferred to a LC vial. In the serotonin analysis, 300 µL of the upper polar phase was transferred via syringe filtration. The sample was then concentrated by centrifugation for 2 hours and freeze-dried for 12 hours. After dissolving in 30 µL of 30% methanol, the sample was transferred to a LC vial.

### LC-MS/MS analytical conditions

The LC-MS/MS analysis was performed using the Nexera UPLC System (Shimadzu, Kyoto, Japan) connected to LCMS-8050 (Shimadzu, Kyoto, Japan) with the ion source of electrospray ionization in the multiple reaction monitoring (MRM) mode^24^. Chromatographic separation was performed using a PFP column (2.1×100 mm, 3 µm, Shimadzu GLC, Tokyo, Japan) at 40 °C with an injection volume of 1 µL. The mobile phases were water with 0.1% formic acid (eluent A) and acetonitrile with 0.1% formic acid (eluent B) with a gradient program: 0.01 minutes, 2% B; 4.0 minutes, 2% B; 6.0 minutes, 80% B; 7.0 minutes, 100% B; 8.3 minutes, 100% B; 8.31 minutes, 2% B; and 10 minutes, 2% B. The flow rate was 0.3 mL/minute with a total run time of 10 minutes. The ion source was operated in the positive ion mode and interface temperatures were set as follows: nebulizer gas: 3.0 L/minute; heating gas: 10 L/minute; drying gas: 10 L/minute; interface temperature: 300 °C; desolvation line temperature: 250 °C; and heat block temperature: 400 °C. MRM conditions are summarized in Table 1. Data acquisition and processing were performed using LabSolutions (Shimadzu, Kyoto, Japan).

**Table 1:** Parameters of MRM transition for neurochemicals and internal standard.

Analyte	Precursor ion (*m/z*)	Product ion (*m/z*)	Q1 Pre Bias (V)	Collision Energy (eV)	Q3 Pre Bias (V)
Glutamate	148.3	84.15	−10	−16	−15
GABA	103.9	87.2	−12	−14	−15
Dopamine	154.3	137.1	−12	−15	−14
Serotonin	177	160.1	−21	−13	−16
Internal standard	123	51	−15	−32	−18

### Data analysis

All statistical analyses were performed using GraphPad Prism version 6.02. The Mann-Whitney U test was used to assess the significance of differences between two independent groups^20^. The Kruskal-Wallis test followed by Dunnett’s multiple comparison analysis was used to assess the significance of differences between three or more independent groups. All data were expressed as a mean ± standard deviation (S.D.). *p* < *0.05* was considered statistically significant.

## Results

### Pan-neuronal *dUbqn* depletion decreases evening activity in *D. melanogaster*

We previously characterized *dUbqn* RNAi flies and found that the knockdown of *dUbqn* in all neurons caused an accumulation of ubiquitylated proteins and affected negative geotaxis in flies^19,20^. The proteostasis impairments caused by *dUbqn* depletion appeared to negatively affect neuronal homeostasis and associated with neurodegeneration. Although a progressive reduction in the neuronal proteostasis capacity is known to generate a normal neuronal aging, it may result in an occurrence of neurodegenerative diseases. Some of the disease developing patients might suffer from a difficulty in sleeping which certainly affects their circadian rhythm^25–27^. Thus, we hypothesized that ubiquilins might be one of the key players in a circadian rhythm alteration and this relationship is worth to investigate.

**Figure 1:**
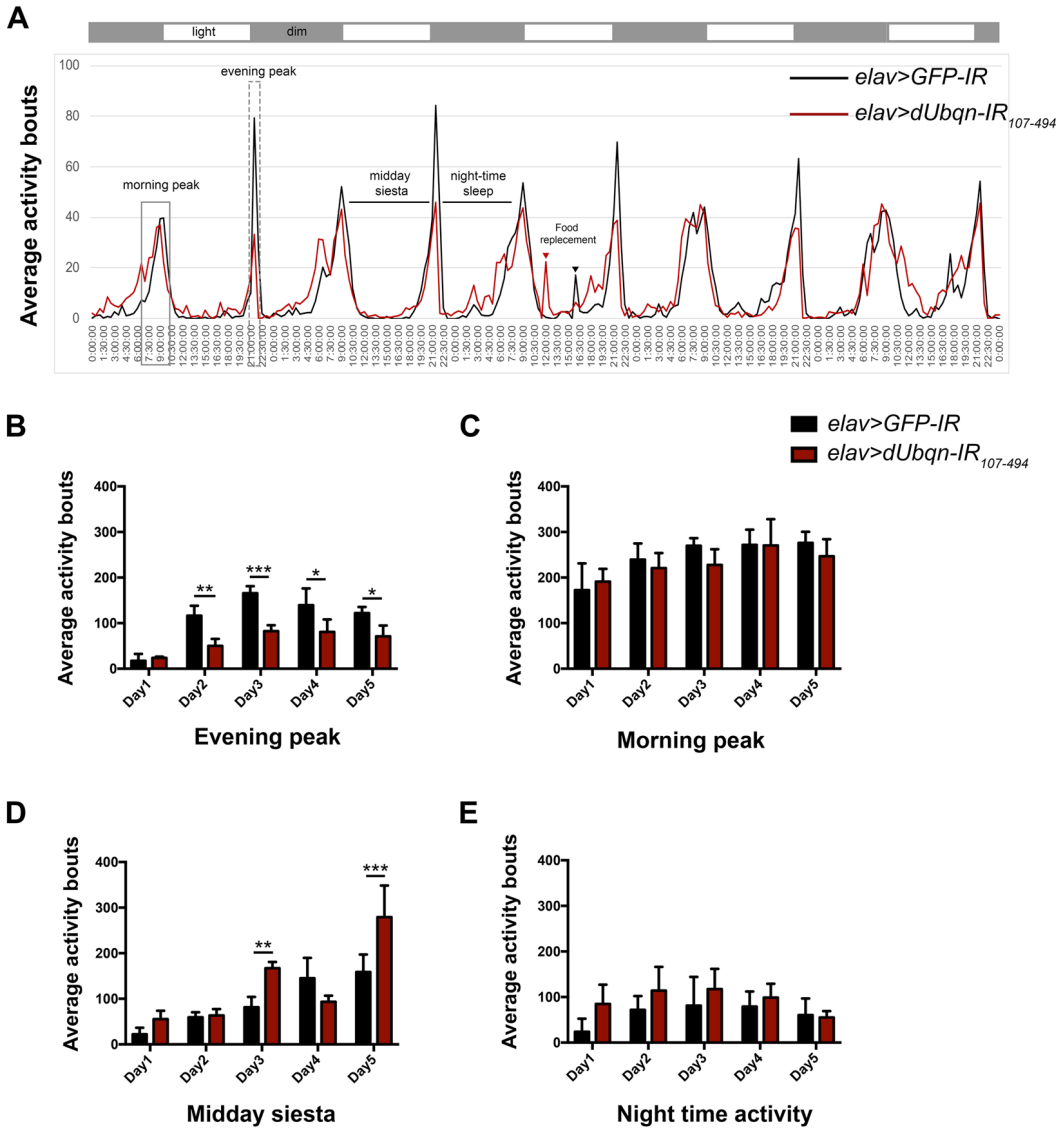
Pan-neuronal knockdown of *dUbqn* reduced *Drosophila* evening activity. (**A)** The daytime and nighttime activity patterns of the following adult flies: *elav* > *GFP-IR* (black line, *w/Y;UAS-GFP-IR*/+;*elav-GAL4*/+, n = 30) and *elav* > *dUbqnIR*_107-494_ (red line, *w/Y;UAS-dUbqnIR*_107-494_/+;*elav-GAL4*/+, n = 30). Grey and white bars indicate 12-h dark and 12-h light periods, respectively. All-day activity bouts are indicated, such as the evening period (Evening peak) (**B)**, morning period (Morning peak) (**C**), afternoon period (Midday siesta) (**D**), and nighttime period (Nighttime activity) (**E**). The experiment was performed in triplicate. Error bars represent the standard deviation (S.D.) of data. *p < 0.05, **p < 0.01, ***p < 0.001.

Due to the fact that, *Drosophila* exhibit daily cycles in their behavioral rhythms that are governed by endogenous circadian rhythms^28^. Therefore, we examined whether the all-day activity or behavioral rhythms of *Drosophila* are disturbed by the knockdown of *dUbqn*. Flies carrying *UAS-dUbqnIR*_107-494_/+;*elav-GAL4*/+ (*dUbqn* depletion) and *UAS-GFP-IR*/+;*elav-GAL4*/+ (control), 3 days after eclosion were used to monitor daily activities by counting the number of times in 30 minutes that each individual fly crossed an infrared light beam. The *dUbqn* RNAi lines with different targeted sequences were used to confirm and minimize the possibility of the off-target effects, as demonstrated previously in various assays^19,20^ and also in the present study (Figs. S1–S3**)**. Both RNAi lines carrying *elav* > *dUbqnIR*_107-494_ and *elav* > *dUbqn-IR*_471-824_ similarly shortened the synapse branch length at NMJ (Fig. S1) and reduced the levels of the dUbqn protein and mRNA to the similar extent (Fig. S2). Control flies showed a regular pattern of activity based on peak activities twice a day between 6.30 to 10.00 am and between 9.00 to 10.00 pm, which are defined as the morning and evening peaks, respectively, and resting activities twice a day between 10.30 am to 6.00 pm and between 10.00 pm to 6.00 am, which are defined as the midday siesta and nighttime sleep, respectively (Fig. 1A).

In comparisons with control flies, *dUbqn*-depleted flies showed a significant decrease in evening activity (evening peak) in the 1–5 days of activity measurements (Fig. 1A,B), but only a slight reduction in morning activity (Fig. 1A,C). On days 3 and 5, we observed a significant increase in activity in *dUbqn*-depleted flies during the resting period, such as the midday siesta, and activity at nighttime also appeared to increase (Fig. 1A,D,E). These results suggest that *dUbqn*-depleted flies exhibited decreased activity, particularly in the evening, which is consistent with our previous findings showing defects in the locomotive activity of *dUbqn*-depleted flies^20^. Another possibility to explain lower evening activity in *dUbqn*-deficient flies is that their higher activity during resting (the midday siesta) might affect their activity in the next period of time (evening); however, further studies are needed to clarify this issue.

### dUbqn plays a role in regulating the presynaptic terminals of the *Drosophila* NMJ

AZ is an important structure for the exchange of chemicals at synapses in which the release of neurochemicals onto postsynaptic receptors is precisely controlled^29,30^. Alterations in AZs have been observed in various *Drosophila* disease models, particularly in neurological disorders, such as ALS, FTD, and ASD^21,31,32^. The immunostaining signals of Bruchpilot (Brp), one of the synaptic proteins located at the AZs of the *Drosophila* NMJs, were attenuated in the *Drosophila* model of ALS and Pitt-Hopkins syndrome^33,34^. Based on previous findings showing defects in the locomotive activity of *dUbqn*-depleted flies^19,20^ and the present results in which flies showed marked reductions in evening peak activity, we hypothesized that reductions in the locomotive activity of *dUbqn*-depleted flies may be caused by alterations in AZs at the presynaptic terminals of the NMJ.

**Figure 2:**
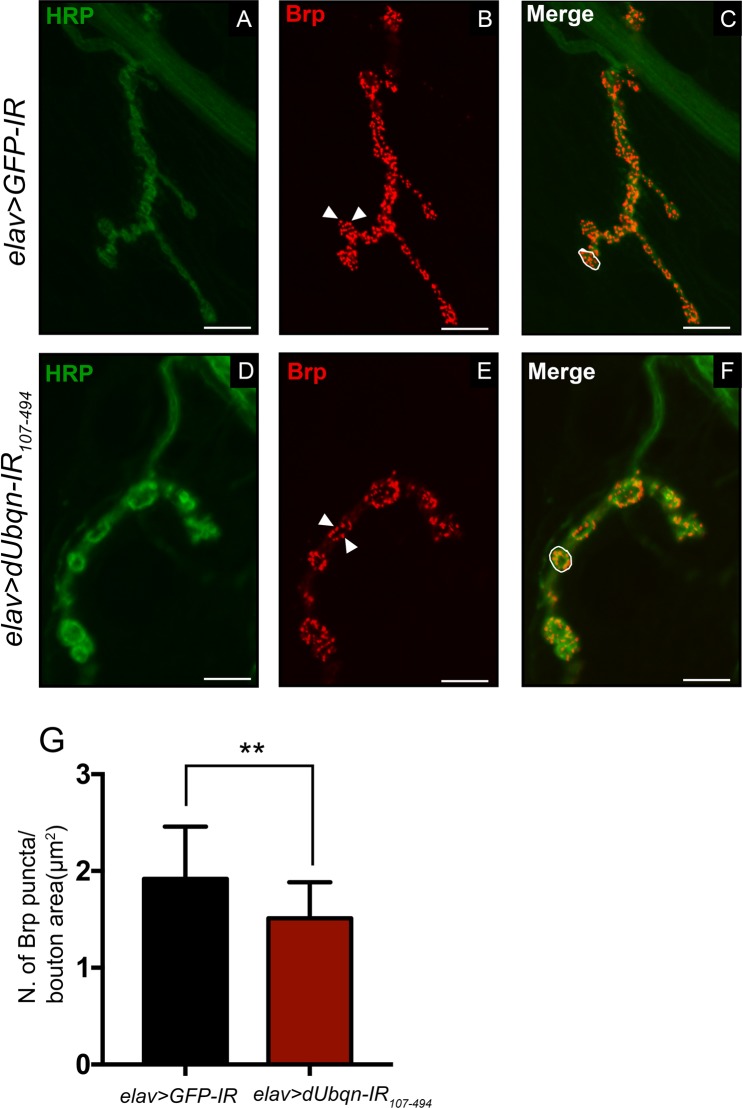
Pan-neuronal knockdown of *dUbqn*-altered *Drosophila* active zones. Confocal micrographs showing the terminal boutons of 4^th^ NMJs from the third instar larvae of (**A**–**C)** *elav* > *GFP-IR (w/Y;UAS-GFP-IR*/+;*elav-GAL4*/+, n = 12) and (**D**–**F)** *elav* > *dUbqnIR*_107-494_ *(w/Y;UAS-dUbqnIR*_107-494_/+;*elav-GAL4*/+, n = 12) immunolabeled with FITC-conjugated anti-HRP IgG (1:1000 dilution) (green) and anti-Brp IgG (1:50 dilution) followed by treatment with Alexa 594-conjugated anti-mouse IgG (1: 400 dilution) (red). Scale bar = 10 μm. (**G)** Quantification of the number of Brp puncta (examples are indicated by white arrowheads) per bouton area (examples are enclosed by white-line). The number of Brp puncta per bouton area was calculated by the total number of “Red spots” divided by the total area of boutons in each NMJ. Error bars represent the standard deviation (S.D.) of data. **p < 0.01.

The majority of adult fly motor neurons originally develop from larval motor neurons^35^. Therefore, we evaluated the expression of Brp by quantifying the density of Brp-positive puncta on NMJs. The Brp puncta were generally distributed throughout control NMJs (Fig. 2A–C), but were reduced in *dUbqn*-depleted NMJs (Fig. 2D–F). Due to the differences in the size and length of NMJs between groups^20^, we decided to quantify the number of Brp puncta by normalization to the related bouton area. We found that the number of Brp puncta was significantly lower in *dUbqn*-depleted NMJs than in control NMJs (Fig. 2G), suggesting that locomotive defects in *dUbqn*-depleted flies might be possibly caused by presynaptic alterations.

Theoretically, neurons compensate for changes in excitatory synaptic outputs to maintain a normal synaptic output^36^. Reductions in Brp expression in the *Drosophila* NMJs may reflect a deficiency in the presynaptic output, and, thus, compensatory processes may take function to optimize a stability of neurotransmission by increasing the highly abundant glutamate receptor (GluR) in the postsynaptic area of the *Drosophila* NMJs^37^. Vice versa, when GluRIIA receptors are reduced, homeostasis retrospectively responds by increasing presynaptic glutamate release from AZs^38,39^. The distribution of GluR in the NMJ can be monitored by one of its subunits, GluRIIA.

**Figure 3:**
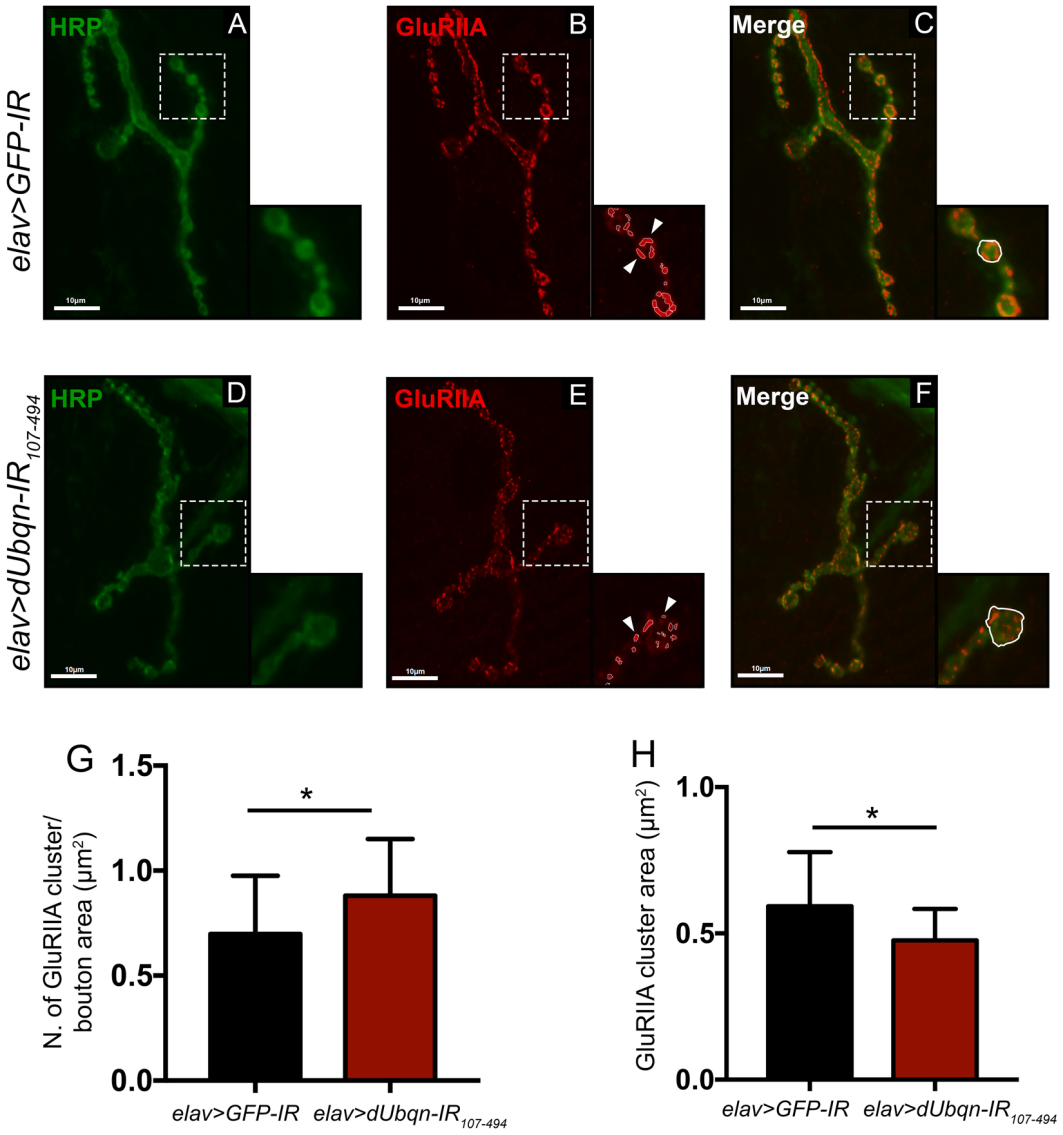
Pan-neuronal knockdown of *dUbqn* altered *Drosophila* post-synaptic glutamate receptor clusters. Confocal micrographs showing the terminal boutons of 4^th^ NMJs from the third instar larvae of *elav* > *GFP-IR (w/Y;UAS-GFP-IR*/+;*elav-GAL4*/+, n = 12) (**A**–**C)** and *elav* > *dUbqnIR*_107-494_ *(w/Y;UAS-dUbqnIR*_107-494_ /+;*elav-GAL4*/+, n = 12) (**D**–**F)** immunolabeled with FITC-conjugated anti-HRP IgG (1:1000 dilution) (green) and anti-GluRIIA IgG (1:80 dilution) followed by treatment with Alexa 594-conjugated anti-mouse IgG (1: 400 dilution) (red). Scale bar = 10 μm. (**G)** Quantification of the number of GluRIIA clusters (examples are indicated by white arrowheads) per bouton area (examples are enclosed by white-line). Number of GluRIIA cluster per bouton area was calculated by the total number of “Red spots” divided by the total area of boutons in each NMJ. (**H)** Quantification of the GluRIIA cluster area. GluRIIA cluster area was measure by an average area of each “Red spot”. All results represent the mean ± standard deviation (S.D.) of data. *p < 0.05. The higher magnification image of indicated area of each panel is also shown.

Therefore, we examined the level of GluRIIA at NMJs in *dUbqn*-depleted larvae. The results revealed that postsynaptic GluRIIA signals normalized to the bouton area were markedly increased at the NMJs of *dUbqn*-depleted larvae (Fig. 3A–G). On the other hand, the GluRIIA cluster area was reduced in *dUbqn*-depleted larvae, possibly reflecting partial homeostatic compensation in *dUbqn* depletion (Fig. 3A–F,H). Since the failure of neuronal compensatory mechanisms has been commonly observed in PD, AD, and other neurogenerative diseases, we speculated that the locomotive defects we found in *dUbqn*-depleted flies were possibly caused by presynaptic alterations together with a failure in synaptic compensation.

### A number of neurochemicals were disturbed in whole bodies of *dUbqn*-depleted flies

Identifying the expression of neurochemicals is critical for interpreting neuronal and behavioral activities^40–42^. Based on the present results, we hypothesized that neurochemical dysregulation may be one of the factors affecting the changes observed in sleep-awake activity and locomotive ability in *dUbqn*-depleted flies. Thus, different kinds of neurochemicals, such as GABA, glutamate, dopamine, and serotonin, have been selected for detection using MSI to examine the distribution patterns of neurochemicals with the combination of LC-MS/MS for the relative quantitation of neurochemical levels.

**Figure 4:**
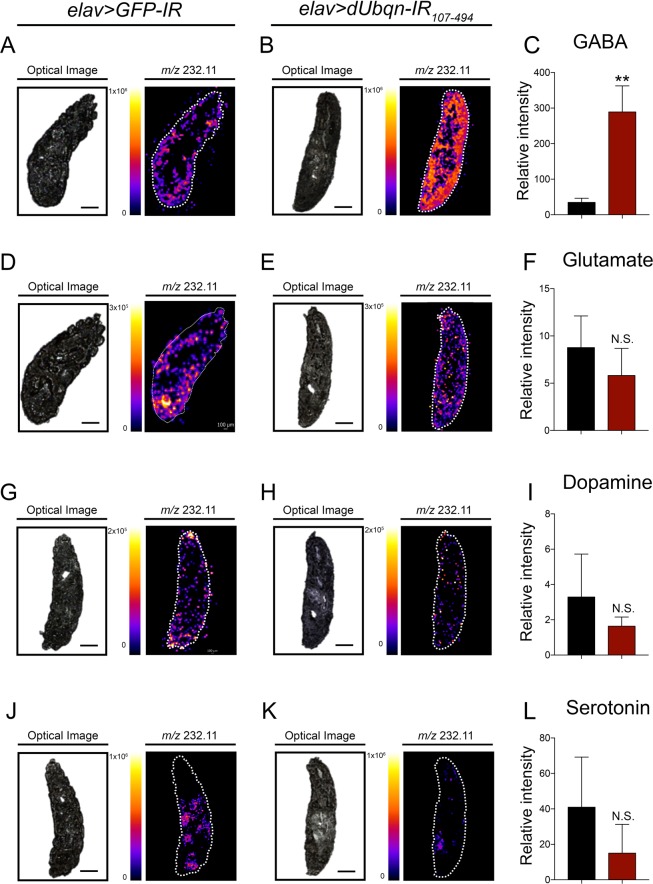
Pan-neuronal knockdown of *dUbqn* disturbs neurochemical distribution. The distribution of GABA, glutamate, dopamine, and serotonin in the whole larval bodies of *elav* > *GFP-IR* (*w/Y;UAS-GFP-IR/+;elav-GAL4/+*, n = 3) (**A**,**D**,**G**,**J**) and *elav* > *UAS-dUbqnIR*_107-494_ (*w/Y;UAS-dUbqnIR*_107-494_ /+;*elav-GAL4*/+, n = 3) (**B**,**E**,**H**,**K**). Optical images are shown on the left and the signal intensity map is shown on the right. White dashed lines indicate the area of larval bodies. Scale bar = 200 μm. Quantification of GABA (**C**), Glutamate (**F**), Dopamine (**I**) and Serotonin (**L**) intensity are shown. All results represent the mean ± standard deviation (S.D.) of data. **p < 0.01 and N.S. = not significant.

Each of the neurochemicals showed a different pattern of distribution under control and *dUbqn*-depleted conditions. GABA and glutamate were detected alongside the larval bodies (Figs. 4A,B,D,E and S3A,B,D,E), whereas dopamine generally diffused throughout the body (Figs. 4G,H and S3G,H). Serotonin showed a pattern that differed from the others due to its dispersal near the internal organs of larvae (Figs. 4J,K and S3J,K). When we quantified the signal intensity of each neurochemical, the data revealed that *dUbqn*-depleted larvae (*elav* > *dUbqn-IR*_107-494_) markedly showed an increase of GABA intensity (Fig. 4C) whereas the other neurochemicals seemed to be declined as comparison to the control (Fig. 4F,I,L). Similarly, we have confirmed the significant decrease in glutamate and dopamine intensity with another *dUbqn* RNAi line (*elav* > *dUbqn-IR*_471-824_) along with a slightly increase and decrease of GABA and serotonin intensity, respectively compared to the control (Fig. S3A–L).

**Figure 5:**
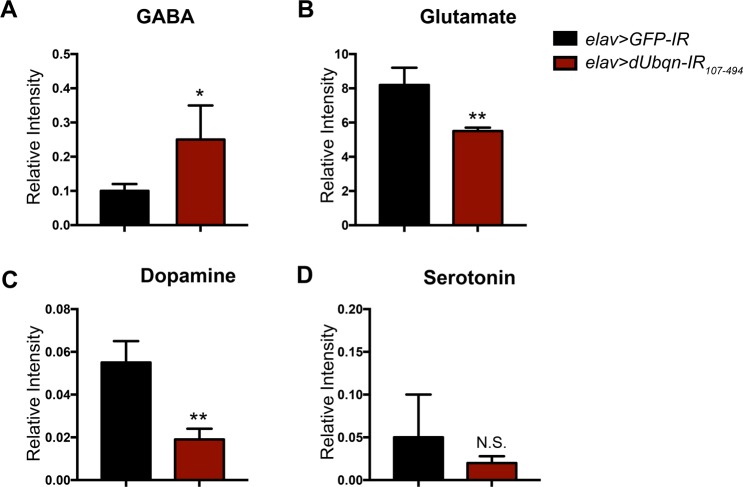
Pan-neuronal knockdown of *dUbqn* changes neurochemical levels. The levels of GABA (**A**), glutamate (**B**), dopamine (**C**), and serotonin (**D**) in the whole larval bodies of *elav* > *GFP-IR (w/Y;UAS-GFP-IR*/+;*elav-GAL4*/+, n = 3–5) and *elav* > *dUbqnIR*_107-494_ *(w/Y;UAS-dUbqnIR*_107-494_/+;*elav-GAL4*/+, n = 3–5) are shown. All results represent the mean ± standard deviation (S.D.). *p < 0.05, **p < 0.01 and N.S. = not significant.

We have also quantified levels of each neurochemical by using LC-MS/MS. Under *dUbqn*-depleted conditions, the level of the inhibitory neurochemical, GABA was significantly higher (Fig. 5A) than under control conditions. The levels of excitatory neurochemicals, such as glutamate and dopamine, were significantly decreased (Fig. 5B,C), while those of serotonin were slightly decreased (Fig. 5D). These results suggested that the dysregulation of neurochemicals might be participated in order to reduce the activity in *dUbqn*-depleted flies.

### The perturbation of courtship behavior was observed in *dUbqn*-depleted flies

*Drosophila* has complex behaviors that are regulated by dopamine and serotonin such as feeding, locomotion, and courtship behaviors^40,41,43,44^. From LC-MS/MS data, we also found reductions in dopamine and serotonin levels under *dUbqn*-depleted conditions. These results prompted us to examine whether *dUbqn*-depleted flies have dysfunctions in a fundamental behavior, such as their courtship behaviors.

**Figure 6:**
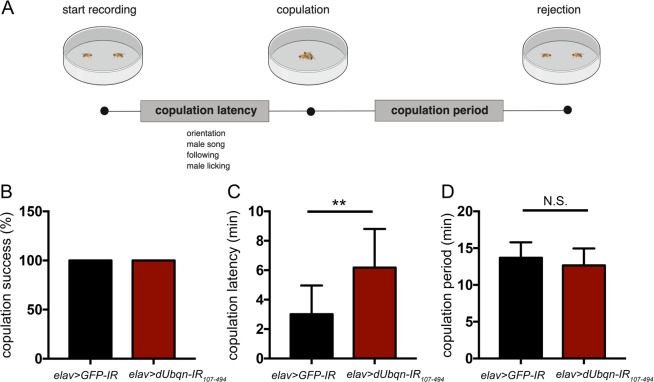
Pan-neuronal knockdown of *dUbqn* causes defects in courtship behaviors. The algorithm of *Drosophila* courtship behaviors shows copulation latency and copulation period (**A**). The percentage of successful copulation (**B**), the copulation latency (**C**), and copulation period (**D**) were compared in flies carrying *elav* > *GFP-IR (w/Y;UAS-GFP-IR*/+;*elav-GAL4*/+, n = 26) and *elav* > *dUbqnIR*_107-494_ *(w/Y;UAS-dUbqnIR*_107-494_/+;*elav-GAL4*/+, n = 26). All results represent the mean ± standard deviation (S.D.). **p < 0.01.

Pairs of 3-day-old virgin flies were used to perform courtship behaviors. Most of the control and the *dUbqn*-depleted flies have completed copulation processes (Figs. 6B and S4A). During copulation latency, the male started orientation behaviors by turning toward and trying to catch the female. The male then tapped the female’s body using his foreleg. The male made wing vibrations, called the “courtship song”, extended his proboscis, and licked the female’s genitalia. Courtship was considered to be successful following acceptance by the female to mounting and the male ejaculating his seminal fluid into the female (Fig. 6A). The time after mounting to separation was generally approximately 10–20 minutes^22^ and we observed approximately 12–17 minutes of mounting in control and *dUbqn*-deficient pairs (Fig. 6D). An alteration of either copulation processes such as an extension of copulation latency or a shortening of copulation period might reveal some defects in courtship behaviors. The *dUbqn*-depleted pairs (*elav* > *dUbqn-IR*_107-494_) had a longer copulation latency of nearly double that of control pairs (Fig. 6C). It means that the affected flies required more time to recognize and respond to mating songs. In addition, a shortening of copulation period was also observed with another dUbqn RNAi line (*elav* > *dUbqn-IR*_471-824_) (Fig. S4C). Cumulatively, our results suggested that an alteration in related neurochemical regulations such as dopamine and serotonin might be one of the causes of courtship behavioral defects in *dUbqn*-depleted flies.

## Discussion and Conclusion

The present results demonstrated that pan-neuronal *dUbqn* depletion, which is known to cause proteostasis impairments, reduced evening time activity and altered pre- and postsynaptic structural regulation in *Drosophila* NMJs. Moreover, the depletion of *dUbqn*, particularly in neurons, dysregulated neurochemicals, likely resulting in sexual behavioral alterations.

We previously reported that *dUbqn*-depleted flies exhibit locomotive defects and aberrant NMJ macrostructures^20^. The *Drosophila* activity monitoring assay, which follows the all-day activity of individual flies over many days, showed the daily rhythms of flies driven by endogenous circadian systems^45^. Consistent with our previous findings, we confirmed a reduction in locomotive activities of *dUbqn*-depleted flies, particularly in the evening peak, whereas normal flies performed peak activities during the same period. Moreover, *dUbqn*-depleted flies showed slightly increased activities during the resting period for many days, suggesting a disturbance in their circadian rhythms.

Brp is an important synaptic protein located in the AZs of neurotransmitter release sites^46,47^. Alterations in AZs have been found in various types of animal models related to neurological disorders, such as ALS, Pitt-Hopkins syndrome, and ASD^21,33,34^. Since *dUbqn*-depleted flies showed locomotive defects^20^, reductions in Brp may reflect synaptic defects in *dUbqn*-depleted flies that already exhibit difficulty with movement.

Homeostatic systems are considered to maintain the stability of neurotransmission. Synapses at the *Drosophila* NMJ are glutamatergic synapses that contain functional GluRIIA, GluRIIB, and other subunits^38,48,49^. Theoretically, the loss of both GluRIIA and GluRIIC subunits triggers a homeostatic increase in the quantal content to maintain sufficient neuronal activity^38,39,48,50^. On the other hand, the observed increase in GluR numbers in the present study may have been triggered by a compensatory mechanism to counteract reductions in presynaptic glutamate levels. Clustered GluRs are normally large and immobilized, whereas newly forming clusters are observed as the diffused form; therefore, reductions in the GluRIIA cluster area in *dUbqn*-depleted NMJs may reflect a sign of postsynaptic developmental defects that ultimately affect fly’s movements.

Similar to humans, a number of neurochemicals are involved in the regulation of *Drosophila* activities and behaviors, such as glutamate, GABA, dopamine, and serotonin^44,51,52^. In the present study, we found a decrease in glutamate, an excitatory neurotransmitter, and increase in GABA, an inhibitory neurotransmitter, resulting in less activity, which is consistent with the results obtained in *dUbqn*-depleted flies. Considering the dopamine which is important for behavioral modulation in *Drosophila* especially an arousal^53^ and courtship behaviors^54^. Previous studies have reported that the *dopamine*-depleted female flies showed an increase in copulation latency whereas D1 dopamine receptor mutants in male flies found a delay in courtship initiation^55,56^. Moreover, we have found that *dUbqn*-depleted flies showed an alteration of dopamine and serotonin regulations that are confirmed the impact of the neurochemicals on sexual behaviors observed in our study. The sexual behavioral defects in humans might be caused by both mental and physical mechanisms. However, due to the limitation of the invertebrates. We cannot clearly justify whether the defects caused by mental or physical problems. Considering our experimental design, the courtship parameters that we have chosen such as the copulation latency and the copulation period, persuaded us to investigate the physical state rather than the mental state. However, the mammalian models are needed for further investigation.

Ubiquitylation is a critical mechanism controlling synaptic plasticity and architecture^57,58^, and its dysregulation has been implicated in numerous neurological disorders and psychiatric diseases^59–61^. Due to the roles of ubiquilins, which are closely related to ubiquitylation, further studies are warranted to clarify the mechanisms by which ubiquilin regulates synapses as well as neurochemicals. Despite the neurochemicals in both central and peripheral nervous system has been detected in our study, an exploration in the specific area is necessary and meaningful. An accumulation of the neurochemicals in some area outside brains might be caused by the specific release of the neurochemicals from the neurons in the peripheral nervous system. However, we cannot exclude the possibility that they are released in the process of preparing tissues, although the signals could be detected only inside larval bodies. Further analysis is necessary to understand the meaning of these observations.

The present study is the first to reveal the role of dUbqn in the regulation of synapses and neurochemicals, and impairments in its function may ultimately affect fundamental activities including locomotive and sexual activities. The *dUbqn*-deficient model with proteostasis impairments may be an important model for identifying early-stage markers of neurological disorders and developing promising therapies in the future.

## Supplementary information

Supplementary Figures.

## Data Availability

All of the original data are available on request.
