# Supplementary material for: The depletion of ubiquilin in Drosophila melanogaster disturbs neurochemical regulation to drive activity and behavioral deficits
Source: Sci Rep. 2020 Mar 30;10:5689. doi: 10.1038/s41598-020-62520-y (PMC7105486; doi:10.1038/s41598-020-62520-y)
Supplement: Supplementary file 1 — Supplementary Figures. [file 41598_2020_62520_MOESM1_ESM.pdf]

The depletion of ubiquilin in *Drosophila melanogaster* disturbs neurochemical regulation to drive activity and behavioral deficits

Salinee Jantrapirom[1,2], Yosuke Enomoto[3], Jirarat Karinchai[4], Mizuki Yamaguchi[2], Hideki Yoshida[2], Eiichiro Fukusaki[3], Shuichi Shimma[3,*], Masamitsu Yamaguchi[2,*]

[1]*Department of Pharmacology, Faculty of Medicine, Chiang Mai University, Chiang Mai, 50200, Thailand*
[2]*Department of Applied Biology, Kyoto Institute of Technology, Matsugasaki, Sakyo, Kyoto 606-8585, Japan*
[3]*Department of Biotechnology, Graduate School of Engineering, Osaka University, 2-1 Yamadaoka, Suita, Osaka 565-0871, Japan*
[4]*Department of Biochemistry, Faculty of Medicine, Chiang Mai University, Chiang Mai, 50200, Thailand*

[*]Double correspondence: MY, myamaguc@kit.ac.jp; SS, sshimma@bio.eng.osaka-u.ac.jp

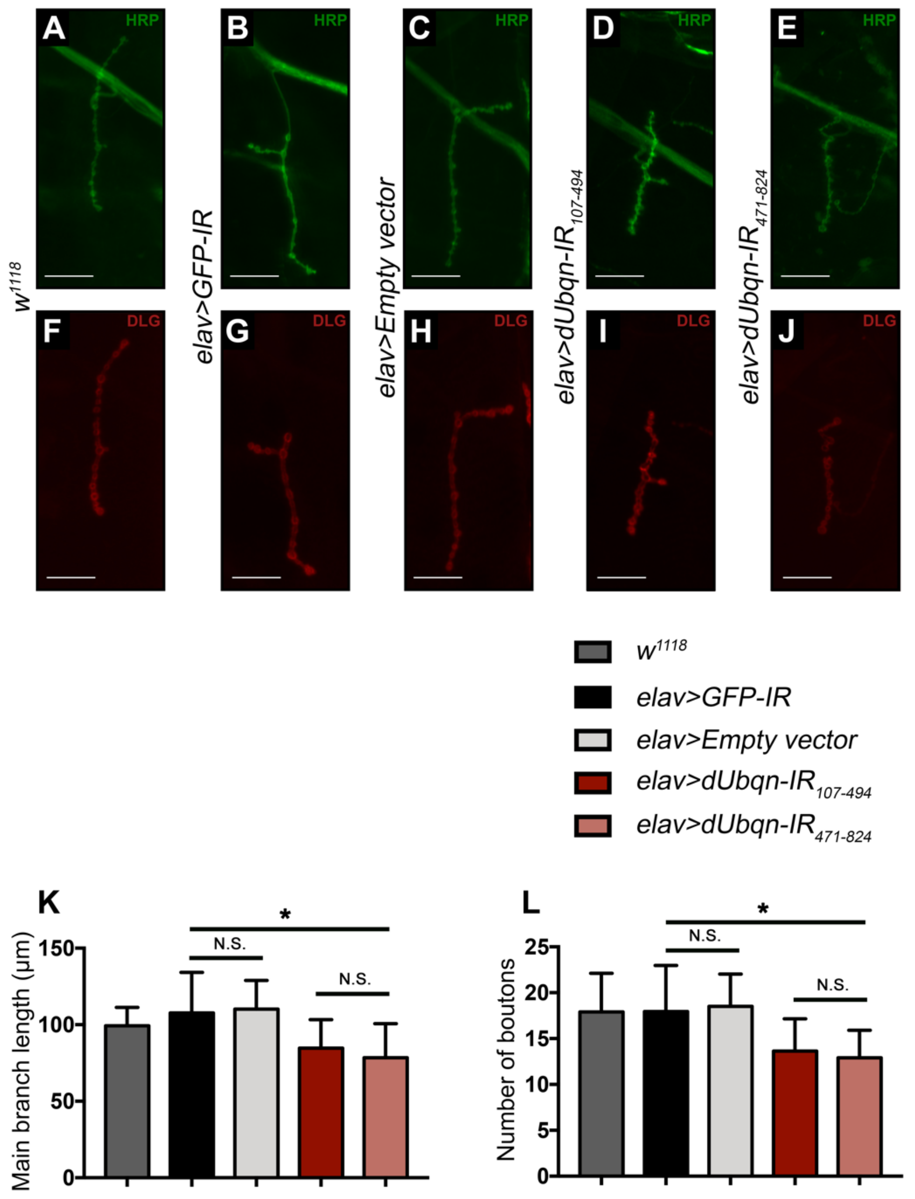

Figure S1 Pan-neuronal knockdown of *dUbqn*-altered *Drosophila* NMJs.
Confocal micrographs showing the terminal boutons of 4th NMJs from the third instar larvae of **A,F)** *w^{1118}*, n=12 **B,G)** *elav-GAL4>GFP-IR (w/Y;UAS-GFP-IR/+;elav-GAL4/+*, n=12) **C,H)** *elav-GAL4>Empty vector* **D,I)** *elav>dUbqn-IR$_{107\text{-}494}$ (w/Y; UAS-dUbqn-IR$_{107\text{-}494}$/+;elav-GAL4/+*, n=12) **E,J)** *elav>dUbqn-IR$_{471\text{-}824}$ (w/Y;+; UAS-dUbqn-IR$_{471\text{-}824}$/elav-GAL4*, n=12) immunolabeled with FITC-conjugated anti-HRP IgG (1:1000 dilution) (green) and anti-Dlg IgG (1:300 dilution) followed by treatment with Alexa 594-conjugated anti-mouse IgG (1: 400 dilution) (red). Scale bar = 10 µm. **G)** Quantification of the main branch length **K)** and number of total boutons of NMJs **L)**. Error bars represent the standard deviation (S.D.) of data. *p < 0.05, N.S. = not significant

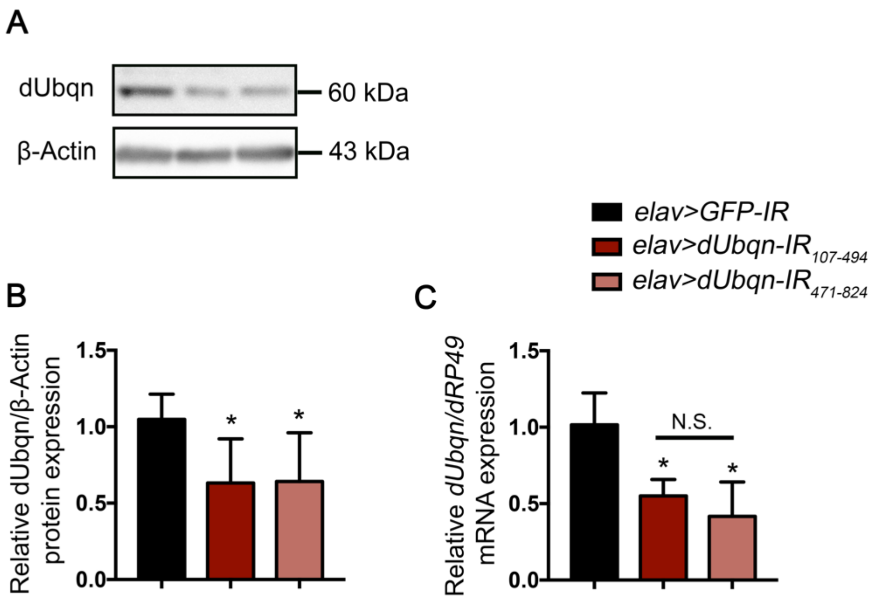

Figure S2 dUbqn protein and it's mRNA expression.
Proteins were extracted from 50 heads of each group of newly-eclosed flies carrying *elav-GAL4>GFP-IR (w/Y;UAS-GFP-IR/+;elav-GAL4/+*, n=3), *elav>dUbqn-IR*$_{107\text{-}494}$ *(w/Y; UAS-dUbqn-IR*$_{107\text{-}494}$*/+;elav-GAL4/+*, n=3) and *elav>dUbqn-IR*$_{471\text{-}824}$ *(w/Y;+; UAS-dUbqn-IR*$_{471\text{-}824}$*/elav-GAL4*, n=3), respectively. Blots were probed with anti-dUbqn IgG and anti-β-Actin IgG **A)**. Quantification of dUbqn was performed by comparing with the abundance of β-Actin **B)**. Total RNAs were extracted from 60 heads of each group of newly-eclosed flies as mentioned. The level of *dUbqn* transcript was measured by normalizing to *dRP49* abundance **C)**. All experiments were done in triplicate. Error bars represent the standard deviation (S.D.) of data. *$p < 0.05$.

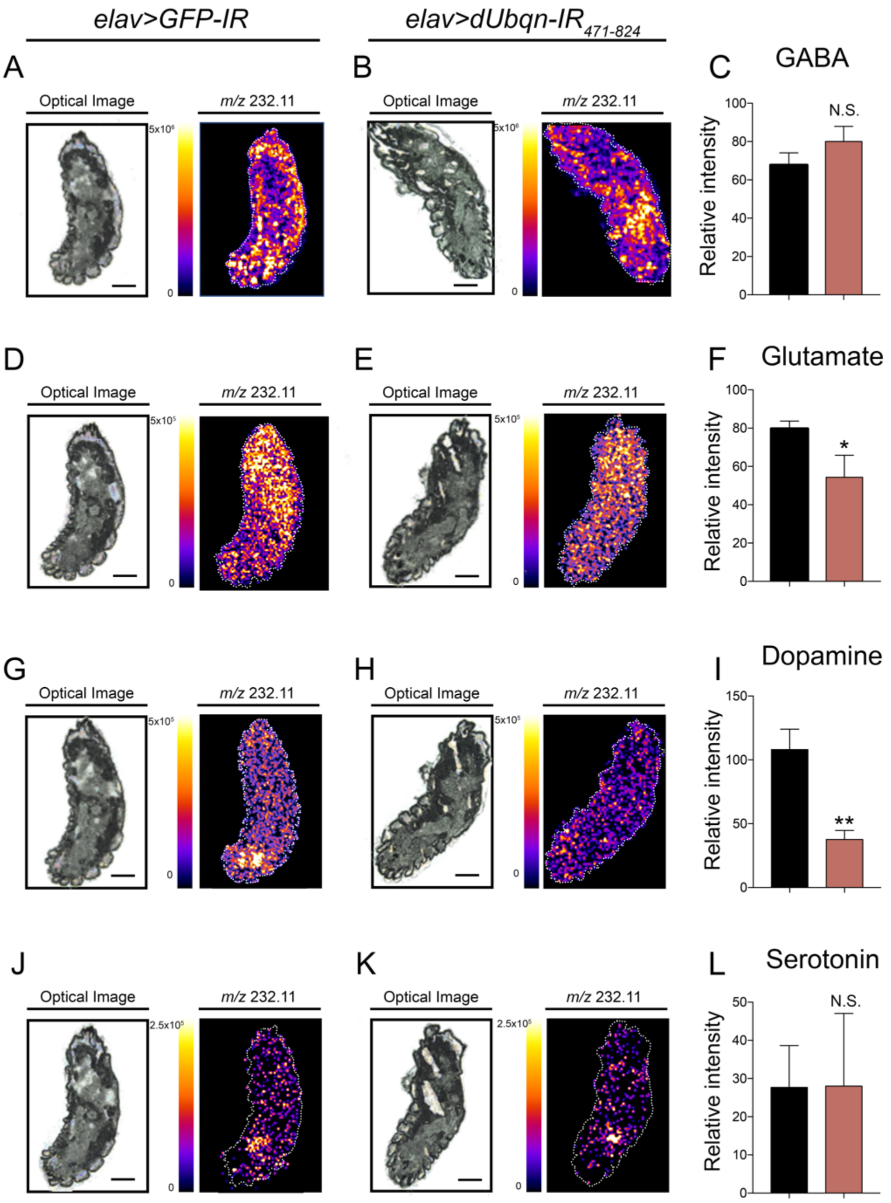

Figure S3 The neurochemical distribution and quantification

The distribution of GABA, glutamate, dopamine, and serotonin in the whole larval bodies of *elav-GAL4>GFP-IR* (*w/Y;UAS-GFP-IR/+;elav-GAL4/+*, n=3) (**A, D, G,** and **J**) and *elav-GAL4>UAS-dUbqn-IR*$_{471\text{-}824}$ (*w/Y;+; UAS-dUbqn-IR*$_{471\text{-}824}$*/elav-GAL4*, n=3) (**B, E, H,** and **K**). Optical images are shown on the left and the signal intensity map is shown on the right. White dashed lines indicate the area of larval bodies. Scale bar = 200 μm. Quantification of GABA (**C**), Glutamate (**F**), Dopamine (**I**) and Serotonin (**L**) intensity are shown. All results represent the mean ± standard deviation (S.D.) of data. $**p < 0.05$, $**p < 0.01$ and N.S. = not significant.

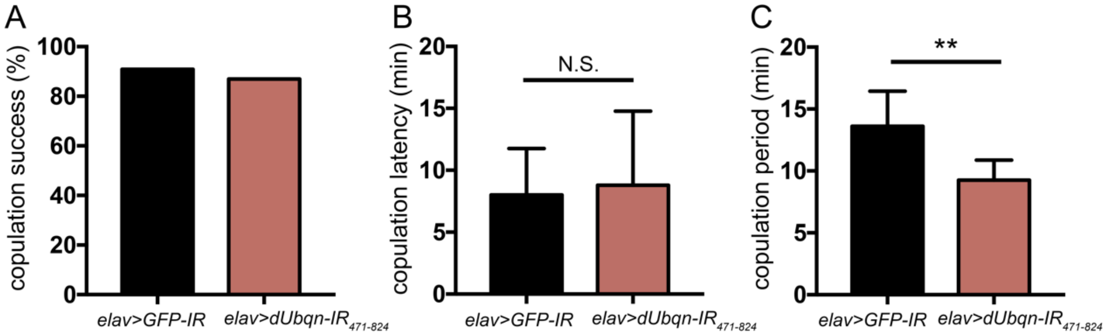

Figure S4 The courtship behavioral measurement

The percentage of successful copulation (**A**), the copulation latency (**B**), and copulation period (**C**) were compared in flies carrying *elav-GAL4>GFP-IR* (*w/Y;UAS-GFP-IR/+;elav-GAL4/+*, n=23) and *elav-GAL4>dUbqn-IR*$_{471\text{-}824}$(*w/Y;+; UAS-dUbqn-IR*$_{471\text{-}824}$/*elav-GAL4*, n=23). All results represent the mean ± standard deviation (S.D.). **$p < 0.01$. N.S. = not significant
